# Comparison of clinical efficacy and surgeon’s neck flexion time between unilateral biportal endoscopic lumbar fusion versus minimally invasive transforaminal lumbar fusion in the treatment of single-level lumbar degenerative diseases: a single center retrospective study

**DOI:** 10.1186/s12891-025-08406-4

**Published:** 2025-03-25

**Authors:** Wen-Bo Wei, Sha-Jie Dang, Da-Peng Duan, Wei Zhao, Ling Wei

**Affiliations:** 1 Department of Orthopedics, Shaanxi Provincial People’s Hospital, Xi’an, Shaanxi China; 2 Shaanxi Province Key Laboratory of Basic and Clinical Translation for Bone and Joint Diseases, Xi’an, Shaanxi China; 3 Department of Anesthesia, Shaanxi Provincial Cancer Hospital, Xi’an, Shaanxi China; 4 Xi’an Jiaotong University, Xi’an, Shaanxi China; 5 Department of Pain, The Third Affiliated Hospital of Xi’an Medical University, Xi’an, Shaanxi 710005 China

**Keywords:** Lumbar degenerative diseases, Unilateral biportal endoscopic lumbar fusion, Minimally invasive transforaminal lumbar fusion, Neck pain

## Abstract

**Purpose:**

This retrospective study aimed to evaluate the clinical efficacy and surgeon’s neck flexion time between unilateral biportal endoscopic lumbar fusion (UBE-LIF) versus minimally invasive transforaminal lumbar fusion (MIS-TLIF) in the treatment of single-level lumbar degenerative diseases (LDD).

**Methods:**

This study retrospectively enrolled patients with single-level LDD received UBE-LIF or MIS-TLIF between June 2018 to May 2022. The patients were separated into two groups based on the surgical method used: the UBE-LIF group (*n* = 38) and the MIS-TLIF group (*n* = 42). Various parameters, including operative time, fluoroscopy frequency, blood loss, length of hospital stay, total expenses, visual analogue scale (VAS), and Oswestry Disability Index (ODI), modified MacNab criteria, fusion rate, and complications were evaluated and compared between the two groups. And time for neck flexion during surgery by the surgeon were recorded. After the surgery, surgeons completed a questionnaire based on a visual analog scale to assess their discomfort symptoms of the neck, shoulders, and back.

**Results:**

There were no significant differences in VAS or ODI scores at 12 months after surgery among two groups. However, the UBE-LIF group had significantly better VAS scores for low back pain on the first day after surgery than the MIS-TLIF group(2.00 ± 0.70) (2.55 ± 0.94) (*P* < 0.05). Additionally, the UBE-LIF group had shorter intraoperative bleeding (78.42 ± 51.440) ml (169.29 ± 52.656) ml (*P* < 0.05). There were no significant differences in fluoroscopy frequency and incidence of complications among two groups. But total expenses in the UBE-LIF group (73246 ± 4354) yuan were significantly higher than those in the MIS-TLIF group (60577 ± 4160) yuan (*P* < 0.05). In the UBE-LIF group, the surgeon’s neck flexion time was significantly reduced(52.00 ± 18.233) min (102.83 ± 11.77) min (*P* < 0.05), and there was a statistically significant reduction in the visual analog scale discomfort scores for the neck, back, and shoulders (*P* < 0.05).

**Conclusions:**

Both UBE-LIF and MIS-TLIF can achieve good postoperative results. UBE has less intraoperative bleeding, less postoperative drainage flow, lower postoperative patient low back pain score, and slightly higher cost than MIS-TLIF. However, the surgeon has a shorter time to lower their head during surgery and higher comfort during surgery.

Lumbar degenerative diseases(LDD) often occurs in middle-aged and elderly patients, including lumbar disc herniation (LDH), lumbar spinal stenosis, lumbar spondylolisthesis [1]. LDD often cause low back pain and sciatica, which seriously impact patient’s life. For patients with ineffective conservative treatment, surgical treatment is required [2]. Traditional open surgery have drawbacks, consists of substantial perioperative muscle dissection, significant blood loss, and protracted postoperative recovery periods [3].Advancements in technology and the maturation of minimally invasive surgical approaches have led to procedures like minimally invasive transforaminal lumbar fusion (MIS-TLIF), which mitigate iatrogenic injuries to soft tissues, including the paraspinal muscles, reduced trauma, diminished blood loss, and accelerated postoperative recovery [4]. Currently, MIS-TLIF has emerged as a standard surgical modality for the management of lumbar degenerative diseases [5].However, complications like ischemia and denervation of the paravertebral muscles due to continuous retractor retraction, poor visualization due to intraoperative tissue bleeding and metal channel obstruction, blood loss, and cavitation may increase the infection rate, and all these issues cannot be ignored [6, 7].

In recent years, water-mediated channel lumbar fusion has gradually become the focus of spine surgeons. Unilateral biportal endoscopic lumbar fusion(UBE-LIF), as a novel minimally invasive fusion surgery, utilizes dual working channels and is executed within a liquid environment. UBE-LIF offers several benefits, including minimal disruption to the multifidus muscle, minimal bleeding, and a clear surgical field [8].

Neck pain and the occurrence of cervical radiculopathy/myelopathy are prevalent issues within the spine surgeons.According to Szeto’ research, when the head is in forward flexion posture for a longer time period, it results in loss of cervical lordosis and potential increase in thoracic kyphosis [9]. Forward head posture combined with increased biodynamic stress of the spine leads to musculoskeletal problems, such as neck pain and headache [10]. The elevated occurrence rates among spine surgeons may be expected from sustained neck flexion during prolonged manual procedures [11, 12]. During UBE surgery, the surgeon predominantly focuses on the monitor and reduce bowing time, potentially enhancing neck comfort.

Currently, there is a notable dearth of comparative analysis between these two surgical approaches, particularly concerning the duration of the surgeon’s neck flexion time. The primary objective is to assess the effectiveness of both minimally invasive surgical procedures and the duration of neck flexion time among surgeons. This evaluation aims to elucidate the strengths and weaknesses inherent in these two surgical methods for single-level pathologies.

## Methods

### Study design

A retrospective review was undertaken, involving the medical records of 80 patients who were treated at the Department of Orthopedics, Shaanxi Provincial People’s Hospital, from June 2018 to May 2022. The study obtained approval from the Clinical Research Ethics Committee of Shaanxi Provincial People’s Hospital (Approval No. 2020-026) and adhered to the guidelines of Good Clinical Practice, as well as the principles outlined in the Helsinki Declaration. The study encompassed 42 cases that underwent MIS-TLIF (Group T) and 38 cases that underwent UBE-LIF (Group U).

### Patients

LDD Patients undergoing MIS-TLIF or UBE-LIF were screened in this study. The surgeon explained the advantages and disadvantages of the two surgical methods and the possible complications to the patients before the operation, and the patients chose the surgical method. All operations were performed by two surgeons, with more than 10 years of spine surgery experience. The inclusion criteria were: (1) single-level lumbar degenerative diseases, including single-level lumbar disc herniation, lumbar spinal stenosis, Grade 1 to 2 degenerative spondylolisthesis, Modic change and iatrogenic lumbar instability; (2) ineffective response to conservative treatment for 3 months; (3) patients experiencing low back pain and/or sciatica, (4) confirmation of signs and symptoms through MRI and CT imaging, (5) underwent MIS-TLIF or UBE-LIF, and (6) at least one year of postoperative follow-up. Exclusion criteria encompassed: (1) Grade 3 or above degenerative; (2) skin or deep-tissue infection in the surgical area; (3) individuals with psychosis or uncorrectable bleeding tendencies; (4) history of previous lumbar surgery; (5) incomplete radiological or treatment information.

### Procedures

Guided by C-arm fluoroscopy, the patient is positioned in the prone position with U-shaped pillows under the chest and both ilia so that the abdomen is suspended.

### UBE-LIF

**Fig. 1:**
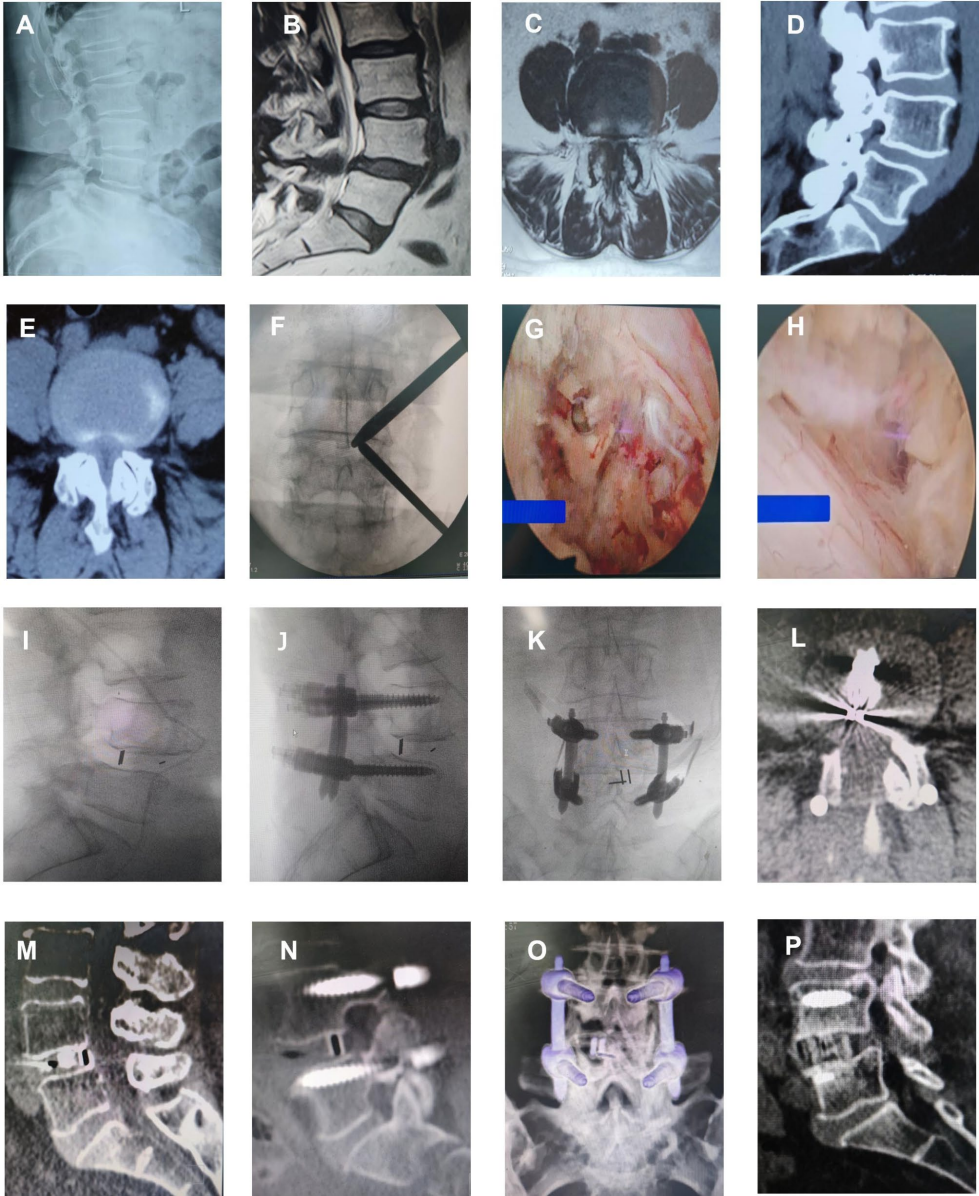
Female, 61 years old, L4/5 degenerative lumbar spinal stenosis. **A**. preoperative lateral X-ray image; **B**. preoperative sagittal MR image; **C**. preoperative axial MR image; **D**. preoperative sagittal CT image; **E**. preoperative axial CT image; **F**. Intraoperative fluoroscopic confirmation of metal rods; **G**.an appropriate size interbody fusion cage was placed under endoscopy; **H**. complete decompression of contralateral recess; **I**. lateral X-ray of intraoperative implantation of fusion cage;**G**. intraoperative lateral X-ray image; **K**. intraoperative anteroposterior X-ray image; **L**. postoperative axial CT image; **M**-**N**. 1 month postoperative sagittal CT image; **O**. 1 month postoperative 3D-CT image; **P**. 12-months postoperative sagittal CT image

After general anesthesia and disinfection of the operation area, two 1-cm incisions were 1.5 cm in the side to the midline and 3 cm apart from each other, which were centered on the target intervertebral space. The two guide rods were inserted through the incision and met at the junction of the upper vertebral lamina and the lower articular process, which was confirmed by fluoroscopy. Then, a T-shaped dilator was used to perform blunt relaxation of the soft tissues. The cranial portal(observational channel) inserts the endoscope (Stryker, Kalamazoo, MI, USA) for view, and the caudal portal working channel is used for surgical instruments and radiofrequency (RF) ablation (BONSS, JiangSu, China). RF ablation and pituitary forceps were used to clean the soft tissue in the field of vision, and expose the upper vertebral lamina and the articular process. Partial laminotomy was performed using a grinding drill (Xishan, Tianjin, China) and Kerrison punches to expose the attachment of ligamentum flavum. The lower edge of the ipsilateral upper vertebral lamina and inferior facet joint were resected using a osteotome, drill, or Kerrison punch. The lateral recess and nerve root canal were decompressed using a high-speed drill and Kerrison punch. For patients with bilateral stenosis, ULBD (Unilateral laminectomy for bilateral decompression) technique were used [13], the lamina bone was removed along the spinous process root to the contralateral side, and to the contralateral facet joint and lateral recess for contralateral decompression. The ligamentum flavum was removed using Kerrison punches. Then expose the dura mater and nerve roots in the spinal canal, the traverse nerve root was gently retracted towards the midline of spinal canal, expose the intervertebral space. The intervertebral space was then processed with a conventional TLIF instrument reamer, pituitary forceps, and curette, and the cartilage endplate was curetted under endoscopic direct vision. Autologous bone and allografts were used for interbody bone grafting through an infundibular bone graft device, followed by endoscopic placement of an interbody cage (PEEK, Sanyou, Inc., ShangHai, China) of appropriate size. then, percutaneous pedicle screw fixation was performed. Finally, C-arm fluoroscopy was performed to confirm the final position of the screw, and a drainage tube was placed before suturing the skin [14].(Fig. 1).

### MIS-TLIF

**Fig. 2:**
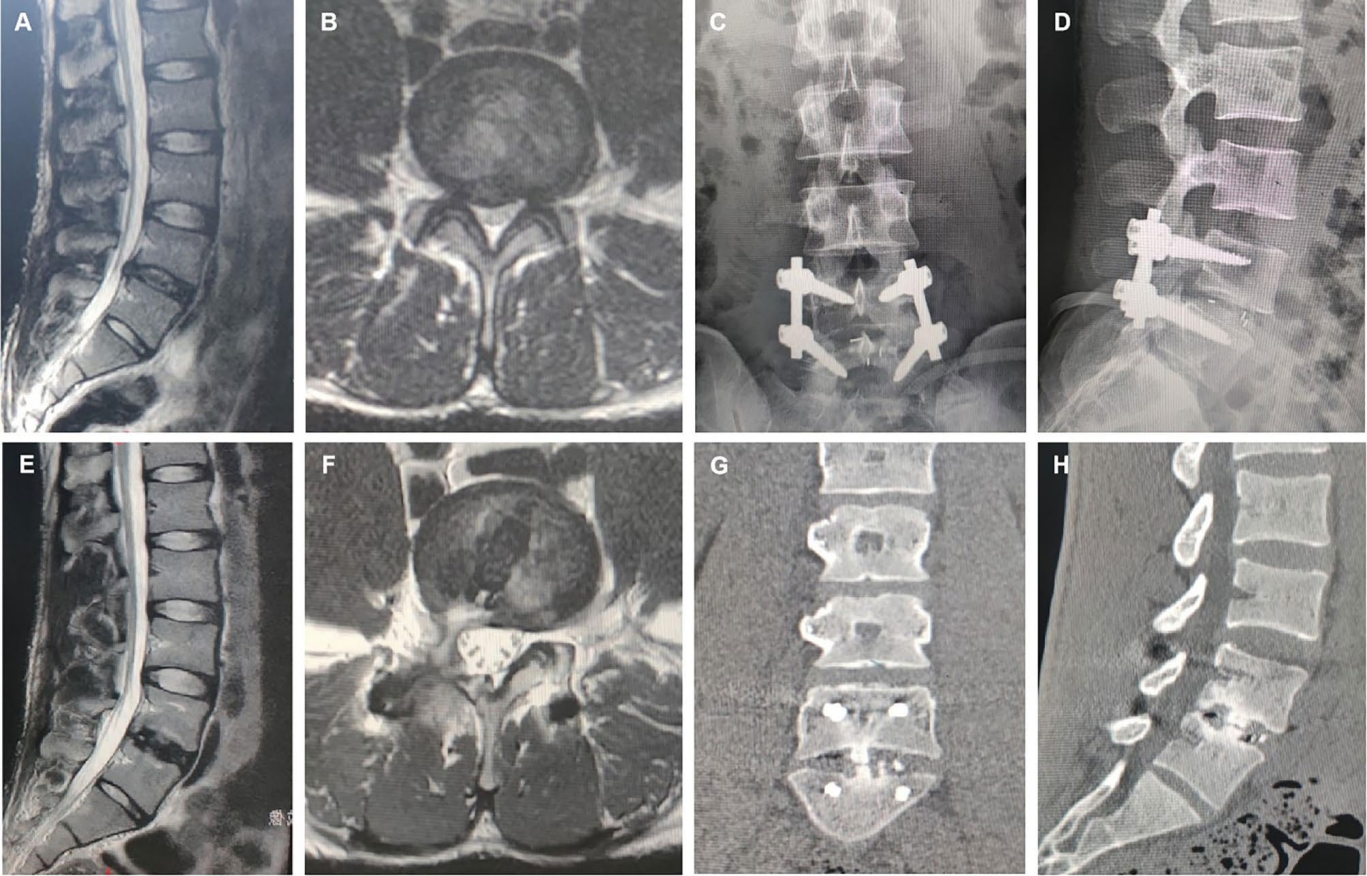
Male, 65 years old, L4/5 degenerative lumbar disc herniation. **A**. preoperative sagittal MR image; **B**. preoperative axial MR image; **C**. postoperative lateral X-ray image; **D**. postoperative anteroposterior X-ray image; **E**. postoperative sagittal MR image; **F**. postoperative axial MR image; **G**. 12-months postoperative coronal CT image; **F**. 12-months postoperative sagittal CT image

After general anesthesia, the patient was placed in a prone position. The target intervertebral space was identified with C-arm fluoroscopy and marked. One 3-cm incision was 1.5 cm in the side to the midline in the target intervertebral space. Then, the Quadrant retractor system was inserted through the Multifidus and Longissimus space. A light source was connected to fully expose the articular process and lamina. Under direct visualization, the Kerrison punch or osteotome was used to remove part of the upper and lower articular processes and part of the lamina. The ligament flavum was then removed and the nerve root and dural sac were sufficiently decompressed. The diseased disc was removed and the compressed nerve root was released. After careful treatment of the endplate, autologous and allografts were used for interbody bone grafting. Then, a standard PEEK interbody cage (Sanyou, Inc., ShangHai, China) were implanted in the intervertebral space. Subsequently, C-arm fluoroscopy confirmed the final location of the cage. Finally, percutaneous pedicle screw fixation was performed and drainage was placed before suturing the wound [15].(Fig. 2).

### Postoperative treatment

If the postoperative drainage fluid is less than 50 ml, the drain was removed. All patients were instructed to wear waist protection after surgery when moving out of bed. After one month of surgery, patients were instructed to wear a lumbar brace and engage in moderate lower back muscle function exercises.

### Outcomes

One doctor recorded the data of two groups of patients. The visual analog scale (VAS) and the Oswestry Disability Index (ODI) scores were measured during pre-operation, 1 day, 1, 3, 6 and 12 months after the operation, respectively. The VAS score was measured on a 10 cm scale, where 0 indicates no pain, and 10 indicates the most severe pain. The ODI assesses low back pain-related disability, the higher the score means the worse the disability. Surgeon’s neck flexion time during surgery were recorded. Neck flexion angle is defined as the angle subtended between vector pointing from C7 to tragus (which corresponds to occipital-cervical joint) and a global vertical line [16]. According to Bigham’s research, staying in extreme neck flexion (> 30°) for extended periods of time during surgery can potentially lead to acute and chronic neck pain [17].We require the circulating nurse to observe every minute whether the surgeon’s neck flexion angle is flexed by more than 30 degrees, and record the flexion time. After the operation, the surgeon scored his neck, shoulder and back joint discomfort on a VAS scale.

The secondary outcomes included several factors: fluoroscopy frequency, in-bed time, blood loss, total expenses, operation time, hospitalization time, and complication. Postoperative blood loss was determined using postoperative drainage volume, and intraoperative blood loss was determined using aspirator suction, irrigation, and lavage volumes. The modified MacNab score at one year was used to evaluate patient satisfaction. Perioperative complications were recorded. At one year, radiography and computed tomography (CT) were performed, and fusion rates were assessed by two radiologists according to the Bridwell grading system, with grades I and II de ned as spinal fusion [18].

### Statistical analysis

SPSS 24.0 for Windows (SPSS, Inc., IBM) was used for statistical analysis. Continuous variables are displayed as the means ± standard errors of the means. Demographic characteristics and perioperative outcomes were assessed via independent sample T-tests, Fisher’s exact tests, or Mann‒Whitney U tests. The ODI and VAS score for back and leg pain were compared via repeated measures analysis of GEEs. A P value less than 0.05 was considered to indicate statistical significance.

## Results

### General information

In total, 80 patients with LDD (aged 45 ~ 78 years old, BMI 16 ~ 38, and ASAI ~ III) were included in our study. No statistically significant differences were noted between the two groups in terms of sex, age, body mass index (BMI), and classification of disease (*P* > 0.05). However, the UBE-LIF group had shorter in-bed time, blood loss, and Hospitalization time, but long operation time and higher total expenses (*P* < 0.05). There were no significant differences in fluoroscopy frequency and incidence of complications among the two groups.(Table 1).

**Table 1:** Comparison of general data between MIS group and UBE group

	Group MIS (*n* = 42)	Group UBE (*n* = 38)	t/$\:{(\text{x}}^{2})$	*P*
Male/female	18/22	16/24	0.205	0.65
Age (years)	59.86 ± 11.33	62.45 ± 8.87	-1.13	0.638
BMI (kg/m^2^)	27.57 ± 3.60	27.14 ± 4.26	0.146	0.835
disc herniation classification				
L2/3	2	2		0.725
L3/4	5	4		
L4/5	25	25		
L5/S1	10	7		
Hospitalization time (days)	13.40 ± 2.49	11.82 ± 3.06	2.55	0.013
Operation time (min)	112.50 ± 12.65	123.42 ± 22.75	-2.686	0.009
Complication	1(%)	1(2.6%)	0.005	0.94
total expenses(yuan)	60,577 ± 4160	73,246 ± 4354	-13.304	< 0.001
fluoroscopy frequency	15.81 ± 2.003	15.89 ± 1.928	-0.193	0.847
Blood loss in operation (ml)	169.29 ± 52.656	78.42 ± 51.440	7.792	< 0.001
Postoperative drainage (ml)	87.14 ± 27.287	26.32 ± 15.496	11.917	< 0.001
Surgeon’s neck flexion time (minutes)	102.83 ± 11.77	52.00 ± 18.233	14.953	< 0.001

**Notes**: Numeric data were expressed as Mean ± SD and analyzed by Independent-Samples T-test. Categorical data were expressed by the number of patients (%) and were analyzed with the χ2 test. Group MIS: MIS-TLIF group; Group UBE: UBE-LIF group. *P < 0.05, Group MIS vs. Group UBE

**Abbreviations**: BMI, body mass index; MIS-TLIF, minimally invasive transforaminal lumbar fusion; UBE-LIF, unilateral biportal endoscopic lumbar fusion

However, surgeon’s neck flexion time in the UBE-LIF group were significantly shorter than those in the Mis-TLIF group (*P* < 0.001). The surgeons’ evaluation of the discomfort level at the end of the operation showed a statistically significant reduction in the VAS score for the neck (*P* = 0 0.037), shoulders (*P* = 0.029), and back (*P* = 0 0.018) in the UBE-LIF group.(Table 2).

**Table 2:** Comparison of VAS for surgeons’ discomfort

Site of Discomfort	Group MIS (*n* = 42)	Group UBE (*n* = 38)	*P*
Neck	5.13 ± 0.47	1.94 ± 0.86	0.037
Shoulders	4.16 ± 1.08	1.24 ± 0.61	0.029
Back	3.84 ± 0.92	1.64 ± 0.46	0.018

**Notes**: Statistical significance was set at *p* < 0.05. Group MIS: MIS-TLIF group; Group UBE: UBE-LIF group

None of the patients in any of the two groups experienced severe complications such as spinal injury or paraplegia. In Group MIS, nerve root injury was observed in one patient, whereas within Group UBE, dural puncture was observed in one patient. The complications were reversible and resolved within 1 month. There was no significant difference in the complication rate during the follow-up period between the two groups (*P* > 0.05) (Table 1).

### Comparison of VAS

There was no significant difference in the postoperative low back pain and sciatica (VAS score) during the pre-operation between the two groups.

The Visual Analog Scale (VAS) scores for low back pain and sciatica significantly decreased at each post-operative observation point when compared to the pre-operative scores in both groups (*p* < 0.05). On the first day following surgery, group MIS-TLIF exhibited higher levels of lower back pain compared to group UBE-LIF (*p* < 0.05). However, There was no statistical difference in VAS between the two groups at 1, 3, 6, and 12 months of the post-operation (*P* > 0.05). (Table 3)

**Table 3:** Comparison of VAS between group MIS and group UBE at different time

Group		Pre-operation	Post-operation				
			1 day	1 month	3 months	6 months	12 months
Group MIS(*n* = 42)	low back pain	7.88 ± 0.80	2.55 ± 0.94^a^	1.95 ± 0.79^a^	1.71 ± 0.67^a^	1.19 ± 0.71^a^	0.76 ± 0.66^a^
	sciatica	7.83 ± 0.98	2.24 ± 0.69^a^	1.69 ± 0.87^a^	1.48 ± 0.74^a^	1.19 ± 0.71^a^	0.66 ± 0.65^a^
Group UBE (*n* = 38)	low back pain	7.63 ± 0.79	2.00 ± 0.70^ab^	1.87 ± 0.67^a^	1.68 ± 0.66^a^	1.18 ± 0.83^a^	0.58 ± 0.60^a^
	sciatica	7.47 ± 0.98	2.37 ± 0.82^a^	1.89 ± 0.98^a^	1.58 ± 0.79^a^	1.18 ± 0.83^a^	0.57 ± 0.59^a^
Time F, *P*	low back pain	1026.035, < 0.001					
	sciatica	757.830, < 0.001					
Group F, *P*	low back pain	12.977, 0.529					
	sciatica	18.528, 0.564					
Time * Group F, *P*	low back pain	1.264, 0.289					
	sciatica	0.974, 0.439					

**Notes**: Data are presented as mean ± SD. The groups were compared by repeated measures analysis of variance (ANOVA). Bonferroni correction was used to correct multiple comparisons. Group MIS: MIS-TLIF group; Group UBE: UBE-LIF group; vs. pre-operation in the same group, ^a^*P* < 0.05; vs. Group UBE in the same time, ^b^*P* < 0.05

**Abbreviations**: VAS, visual analog scale; MIS-TLIF, minimally invasive transforaminal lumbar fusion; UBE-LIF, unilateral biportal endoscopic lumbar fusio

### Comparison of ODI

There was no significant difference observed in ODI score at the pre-operation between the two groups. There was no statistical difference in ODI between the two groups at 1, 3, 6 and 12 months of the post-operation (*P* > 0.05). (Table 4)

**Table 4:** Comparison of ODI between group MIS and group UBE at different time

Group	Pre-operation	Post-operation				
			1 month	3 months	6 months	12 months
Group MIS (*n* = 42)	80.95 ± 6.25		21.86 ± 5.11^a^	19.79 ± 5.75^a^	17.31 ± 5.10^a^	10.52 ± 3.84^a^
Group UBE (*n* = 38)	79.84 ± 6.70		21.24 ± 5.31^a^	19.58 ± 5.71^a^	15.87 ± 3.63^a^	10.79 ± 3.80^a^
Time F, *P*	1882.681, < 0.001					
Group F, *P*	0.656, 0.625					
Time * Group F, *P*	26.762, 0.02					

**Notes**: Data are presented as mean ± SD. The groups were compared by repeated measures analysis of variance (ANOVA). Bonferroni correction was used to correct multiple comparisons. Group MIS: MIS-TLIF group; Group UBE: UBE-LIF group; vs. pre-operation in the same group, ^a^*P* < 0.05; vs. Group UBE in the same time, ^b^*P* < 0.05

**Abbreviations**: ODI, Oswestry Disability Index; MIS-TLIF, minimally invasive transforaminal lumbar fusion; UBE-LIF, unilateral biportal endoscopic lumbar fusion

### MacNab criteria and bridwell grading system

According to the modified MacNab criteria, at 12 months, the excellent and good rates were 94.7% and 88.1% in the UBE-LIF and MIS-TLIF groups, respectively, with no significant difference (*P* > 0.05). Bridwell grading was used for interbody fusion. In the UBE-LIF group, there were 30 grade I, 6 grade II, and 2 grade III cases; the fusion rate was 94.7%. In the MIS-TLIF group, there were 31 grade I, 7 grade II, and 4 grade III cases, with a fusion rate of 90.5%. the fusion rates did not differ between the two groups (*P* > 0.05).(Table 5).

**Table 5:** Comparison of follow-up outcomes in MIS group and UBE group

	Group MIS (*n* = 42)	Group UBE (*n* = 38)	*P*
Modified MacNab score			
Excellent	15	15	0.516
Good	22	21	
Medium	3	1	
Poor	2	1	
Fusion at 12 months			
Grade 1	31	30	0.551
Grade II	7	6	
Grade III	4	2	

**Notes**: Statistical significance was set at *p* < 0.05. Bridwell interbody fusion grading system: Grade I is defined as a fusion with remodeling and trabeculae present; Grade II is an intact graft with incomplete remodeling and no lucency present; Grade III is an intact graft with potential lucency at the cranial or caudal end; Grade IV is absent fusion with collapse/resorption of the graft

**Abbreviations**: MIS-TLIF, minimally invasive transforaminal lumbar fusion; UBE-LIF, unilateral biportal endoscopic lumbar fusion

## Discussion

Over the past decade, minimally invasive lumbar interbody fusion has gained widespread popularity as a treatment option for various lumbar spinal disorders [5].At present, there are many surgical methods for minimally invasive spinal fusion [19]. Most of the existing research focuses on the efficacy of surgical methods on patients, and with low attention paid to work-related neck pain of surgeons [12, 20, 21].

This is the first article to compare the efficacy of the Mis-TLIF versus UBE-LIF, and the neck flexion time of the surgeon during the two operations. Our findings indicate that both UBE-LIF and MIS-TLIF procedures can yield favorable postoperative outcomes. UBE-LIF is associated with reduced intraoperative bleeding, lower lumbar VAS scores within the first month post-surgery, decreased surgeon’s time for neck flexion during the procedure, reducted discomfort for surgeon’s neck, back, and shoulders, and marginally higher costs compared to MIS-TLIF.

As one of the gold standards for minimally invasive spinal fusion, Mis-TLIF can reduce damage to the multifidus muscle, stimulate spinal nerves less, and preserve the integrity of the posterior column structure (supraspinal ligament, interspinal ligament, vertebral lamina) of the spine, which can help patients shorten the recovery time and return to normal work faster [22]. However, in a randomized controlled trial comparing Mis-TLIF with traditional lumbar posterior fusion, multifidus muscle injury was mainly observed in patients receiving PLIF. The clinical results evaluated using VAS and ODI showed significant improvement compared to the baseline, without any inter-group differences [23].This indicates that the size of the surgical incision does not affect postoperative pain and disability, the compression time on the muscles is the key factor affecting postoperative lumbar pain and muscle function recovery [21]. Prolonged compression of muscles can lead to muscle denervation and atrophy, leading to postoperative low back pain and poor muscle function recovery [25, 26]. Therefore, reducing the damage to the paraspinal muscles during surgery is crucial. MIS-TLIF technology is performed using a quadrant retracter system, and the surgery is performed under direct vision with channel assistance. To ensure the surgical range, excessive stretching of the paraspinal muscles during the contralateral decompression process is usually inevitable and can easily lead to local muscle ischemia and postoperative low back pain. Our results showed that the Mis-TLIF group still had residual low back pain on the first day after surgery, which was related to the compression of the muscle by the channel.Reducing excessive opening of the channel during surgery and appropriately relaxing the channel can reduce muscle damage.

UBE technology has emerged as one of the swiftly advancing innovations in the past years [25]. This technology allows for the exchange of observation and operation channels during surgery, avoiding the drawbacks of limited field and operation range of single-channel endoscopy. The two channels of UBE do not interfere with each other, providing high flexibility and a wide field of view, which can effectively reduce traction and injury of the paraspinal muscles, thereby reducing postoperative low back pain, and avoiding significant muscle retraction and stripping. At the same time, combined with the surgical field magnification function of the endoscope, it can significantly improve the ability to distinguish the articular process and vertebral lamina, thereby reducing excessive damage to these structures and reducing the occurrence of postoperative low back pain [28, 29]. In this study, the UBE-LIF group had significantly lower ODI and VAS scores for low back pain within 1 day after surgery compared to the MIS-TLIF group (*P* < 0.05), indicating that ULIF technology can effectively reduce paraspinal muscle injury and bone tissue destruction, alleviate postoperative low back pain, and accelerate postoperative recovery.

Furthermore, UBE-LIF employs open surgical instruments, leading to heightened surgical efficiency. UBE-LIF can be perceived as a form of endoscopic MIS-TLIF surgery, which is in line with the surgical habits of spinal surgeons and has a short learning curve. According to literature reports, the learning curve of intervertebral foramen endoscopy was around 100 cases, and the learning curve of UBE was around 24 cases [28]. UBE leverages endoscopy to expand the surgical field, resulting in improved visibility and minimizing the risk of procedural errors. The use of radiofrequency and the effect of water pressure during the operation significantly reduced the bleeding during the operation. This study revealed a reduction in bleeding during UBE-LIF surgery. Nonetheless, it’s worth noting that the use of radiofrequency scalpel heads for addressing extraspinal cavity issues and hemostasis during UBE surgery does result in slightly higher costs compared to MIS-TLIF.

The study identified that UBE fusion surgery typically requires more time than MIS-TLIF. This discrepancy can be attributed to the following two primary reasons:1.UBE technology involves a lengthier process for creating an extraspinal cavity compared to MIS-TLIF. 2. it presents increased challenges in adjusting the tool operation angles when working within the intervertebral spaces.However, our research team observed that as proficiency in UBE technology was achieved, the time gap between the two procedures gradually diminished.

The concern that continuous fluid irrigation may lead to the washout of graft material and osteogenic progenitors, including hematoma, at the fusion site, potentially impacting spinal fusion negatively, is indeed a significant issue [29]. However, it is noteworthy that previous literature reports have not indicated a decrease in fusion rates despite this concern [32, 33]. The achievement of intervertebral fusion hinges on a meticulous discectomy and thorough preparation of the endplate to create a larger contact area. Under UBE microscopy, the treatment of intervertebral spaces is more comprehensive, facilitating the generation of high-quality bone grafting beds and ensuring the sufficient placement of bone graft material in front of the intervertebral spaces within the visual field. This advancement significantly enhances fusion efficiency. Furthermore, it is essential to have a substantial amount of bone graft material at the anterior edge of the intervertebral space before inserting the fusion cage. Simultaneously, during the placement of the fusion device, horizontal adjustment as much as possible can help reduce the risk of fusion device collapse and boost the fusion success rate. Compared to the MIS-TLIF group, our study found no appreciable difference in fusion rates.There is currently no occurrence of fusion device loosening due to selected suitable size fusion cage.

Neck pain and cervical radiculopathy/myelopathy are common among spine surgeons [14].In previous studies, a prevalence rate of neck pain over the past 12 months was 59-66.3% among spine surgeons [14]. Neck pain was responsible for stopping work in 17.5% of surgeons, with a median of 3.5 days off work [32]. The high incidence rate of neck pain among surgeons is attributed to the prolonged maintenance of neck flexion during surgical procedures [33]. Surgeon work-related neck pain carries potential societal costs, which encompass both direct and indirect consequences. The direct costs associated with this condition include expenses related to time off work and specific treatments for work-related neck pain. These costs may encompass disability insurance and a reduction in income for affected individuals. Indirect costs include delay in evaluation and management of patients. Previous studies on the merits and drawbacks of surgical protocols has predominantly centered on patient outcomes, with comparatively less consideration given to the impact on the surgeons themselves [34, 35]. Historically, during lumbar spine surgery, surgeons were required to work with their heads in a downward position for extended periods, resulting in discomfort and strain in the neck. Reducing neck flexion time is crucial for relieving neck pain. Through the entire UBE surgical procedure, surgeons are only required to maintain a direct gaze at the display in front of them, eliminating the necessity of prolonged neck flexion. This leads to enhanced neck comfort, representing a notable advantage, particularly for surgeons. In our study, the surgeon ‘s neck flexion time in the UBE group was significantly lower than that in the MIS-TLIF group, which could significantly reduce the incidence of neck pain and be more comfortable for the surgeon.

While our study has produced satisfactory short-term results, it is imperative to acknowledge certain limitations. Firstly, the study design was nonrandomized, which may have introduced sampling bias because it was a single-center retrospective analysis with a relatively small sample size. Secondly, this study’s scope was constrained by the inclusion of a limited range of disease types, such as a single grade of lumbar spondylolisthesis, with the exclusion of severely degenerated segments characterized by almost nonexistent discs. Thirdly, due to the single-center study, the sample size of the surgeon is small, it is not possible to evaluate the correlation between the time of the surgeon ‘s neck flexion and the neck pain. Therefore, future research endeavors should prioritize long-term follow-up and the implementation of multicenter, randomized controlled clinical trials encompassing a more diverse range of lumbar degenerative disorders to advance the principles of evidence-based medicine.

## Conclusions

Both UBE-LIF and MIS-TLIF can achieve good postoperative results. UBE has less intraoperative bleeding, less postoperative drainage flow, lower postoperative patient low back pain score, and slightly higher cost than MIS-TLIF. However, the surgeon has a shorter time to lower their head during surgery and higher comfort during surgery. It can be promoted as a minimally invasive surgery.

## Data Availability

No datasets were generated or analysed during the current study.

## Abbreviations

UBE-LIF: Unilateral biportal endoscopic lumbar fusion
MIS-TLIF: Minimally invasive transforaminal lumbar fusion
ASA: American Society of Anesthesiologists

## References

[1] Ravindra VM, Senglaub SS, Rattani A, et al. Degenerative lumbar spine disease: estimating Global Incidence and Worldwide volume. Global Spine J. 2018;8(8):784–94. 10.1177/2192568218770769. 30560029 10.1177/2192568218770769 PMC6293435

[2] Reid PC, Morr S, Kaiser MG. State of the union: a review of lumbar fusion indications and techniques for degenerative spine disease. J Neurosurg Spine. 2019;31(1):1–14. 10.3171/2019.4.SPINE18915. 31261133 10.3171/2019.4.SPINE18915

[3] Pokorny G, Amaral R, Marcelino F, et al. Minimally invasive versus open surgery for degenerative lumbar pathologies:a systematic review and meta-analysis. Eur Spine J. 2022;31(10):2502–26. 10.1007/s00586-022-07327-3. 35871660 10.1007/s00586-022-07327-3 PMC9308956

[4] Lener S, Wipplinger C, Hernandez RN, et al. Defining the MIS-TLIF: a systematic review of techniques and technologies used by surgeons Worldwide. Global Spine J. 2020;10(2 Suppl):S151–67. 10.1177/2192568219882346. 10.1177/2192568219882346 PMC7263344 32528800

[5] Momin AA, Steinmetz MP. Evolution of minimally invasive lumbar spine surgery. World Neurosurg. 2020;140:622–6. 10.1016/j.wneu.2020.05.071. 32434014 10.1016/j.wneu.2020.05.071

[6] Wu J, Zhang C, Lu K, Li C, Zhou Y. A Novel Inextensible Endoscopic Tube Versus Traditional Extensible Retractor System in single-level minimally invasive transforaminal lumbar Interbody Fusion: a prospective Observation Study. Pain Physician. 2019;22(6):E587–99. 31775412

[7] Fan S, Hu Z, Zhao F, Zhao X, Huang Y, Fang X. Multifidus muscle changes and clinical effects of one-level posterior lumbar interbody fusion: minimally invasive procedure versus conventional open approach. Eur Spine J. 2010;19(2):316–24. 10.1007/s00586-009-1191-6. 19876659 10.1007/s00586-009-1191-6 PMC2899808

[8] Kang MS, Heo DH, Kim HB, Chung HT. Biportal endoscopic technique for Transforaminal Lumbar Interbody Fusion: review of current research. Int J Spine Surg. 2021;15(suppl 3):S84–92. 10.14444/8167. 35027471 10.14444/8167 PMC9421279

[9] Szeto GP, Straker L, Raine S. A field comparison of neck and shoulder postures in symptomatic and asymptomatic office workers. Appl Ergon. 2002;33(1):75–84. 10.1016/s0003-6870(01)00043-6. 11831210 10.1016/s0003-6870(01)00043-6

[10] Fernández-de-las-Peñas C, Alonso-Blanco C, Cuadrado ML, Pareja JA. Forward head posture and neck mobility in chronic tension-type headache: a blinded, controlled study. Cephalalgia. 2006;26(3):314–9. 10.1111/j.1468-2982.2005.01042.x. 16472338 10.1111/j.1468-2982.2005.01042.x

[11] Abolfotouh SM, Alnori O, Choma T, Moore D, Abolfotouh MA. Epidemiology of work-related Neck Pain among Spine surgeons [published online ahead of print, 2022 Dec 23]. Global Spine J. 2022;21925682221148685. 10.1177/21925682221148685.

[12] Epstein S, Sparer EH, Tran BN, et al. Prevalence of work-related Musculoskeletal disorders among surgeons and interventionalists: a systematic review and Meta-analysis. JAMA Surg. 2018;153(2):e174947. 10.1001/jamasurg.2017.4947. 29282463 10.1001/jamasurg.2017.4947 PMC5838584

[13] Ralph,Mobbs. Kevin, minimally invasive unilateral laminectomy for bilateral decompression.[J].Jbjs Essentia Surgica techniques, 2017 10.2106/JBJS.ST.16.00072 10.2106/JBJS.ST.16.00072 PMC6132588 30233944

[14] Heo DH, Son SK, Eum JH, Park CK. Fully endoscopic lumbar interbody fusion using a percutaneous unilateral biportal endoscopic technique: technical note and preliminary clinical results. Neurosurg Focus. 2017;43(2):E8. 10.3171/2017.5.FOCUS17146. 28760038 10.3171/2017.5.FOCUS17146

[15] Garg B, Mehta N. Minimally invasive transforaminal lumbar interbody fusion (MI-TLIF): a review of indications, technique, results and complications. J Clin Orthop Trauma. 2019;10(Suppl 1):S156–62. 10.1016/j.jcot.2019.01.008. 31695275 10.1016/j.jcot.2019.01.008 PMC6823784

[16] Young JG, Trudeau M, Odell D, Marinelli K, Dennerlein JT. Touch-screen tablet user configurations and case-supported tilt affect head and neck flexion angles. Work. 2012;41(1):81–91. 10.3233/WOR-2012-1337. 22246308 10.3233/WOR-2012-1337

[17] Bigham JJ, Chang EK, Sorensen M, Chansky HA, Telfer S. Using Wearable Technology to measure the Association between Neck Posture and Pain during Urologic Open and robotic surgery. J Endourol. 2021;35(11):1710–5. 10.1089/end.2021.0260. 33940950 10.1089/end.2021.0260

[18] Bridwell KH, Lenke LG, McEnery KW, Baldus C, Blanke K. Anterior fresh frozen structural allografts in the thoracic and lumbar spine. Do they work if combined with posterior fusion and instrumentation in adult patients with kyphosis or anterior column defects? Spine (Phila Pa 1976). 1995;20(12):1410–8. 7676341

[19] Xu DS, Walker CT, Godzik J, Turner JD, Smith W, Uribe JS. Minimally invasive anterior, lateral, and oblique lumbar interbody fusion: a literature review. Ann Transl Med. 2018;6(6):104. 10.21037/atm.2018.03.24. 29707553 10.21037/atm.2018.03.24 PMC5900070

[20] Rathbone J, Rackham M, Nielsen D, et al. A systematic review of anterior lumbar interbody fusion (ALIF) versus posterior lumbar interbody fusion (PLIF), transforaminal lumbar interbody fusion (TLIF), posterolateral lumbar fusion (PLF). Eur Spine J. 2023;32(6):1911–26. 10.1007/s00586-023-07567-x. 37071155 10.1007/s00586-023-07567-x

[21] Heemskerk JL, Oluwadara Akinduro O, Clifton W, Quiñones-Hinojosa A, Abode-Iyamah KO. Long-term clinical outcome of minimally invasive versus open single-level transforaminal lumbar interbody fusion for degenerative lumbar diseases: a meta-analysis. Spine J. 2021;21(12):2049–65. 10.1016/j.spinee.2021.07.006. 34273567 10.1016/j.spinee.2021.07.006

[22] Kim CH, Easley K, Lee JS, Hong JY, Virk M, Hsieh PC, Yoon ST. Comparison of minimally invasive versus open transforaminal interbody lumbar fusion. Glob Spine J. 2020;10:s143–50. 10.1177/2192568219882344. 10.1177/2192568219882344 PMC7263326 32528799

[23] Putzier M, Hartwig T, Hoff EK, Streitparth F, Strube P. Minimally invasive TLIF leads to increased muscle sparing of the multifidus muscle but not the longissimus muscle compared with conventional PLIF-a prospective randomized clinical trial. Spine J. 2016;16:811–9. 10.1016/j.spinee.2015.07.460. 26235468 10.1016/j.spinee.2015.07.460

[24] Min WK, Kim JE, Choi DJ, Park EJ, Heo J. Clinical and radiological outcomes between biportal endoscopic decompression and microscopic decompression in lumbar spinal stenosis. J Orthop Sci. 2020;25(3):371–8. 10.1016/j.jos.2019.05.022. 31255456 10.1016/j.jos.2019.05.022

[25] Kotil K, Tunckale T, Tatar Z, Koldas M, Kural A, Bilge T. Serum creatine phosphokinase activity and histological changes in the multifidus muscle: a prospective randomized controlled comparative study of discectomy with or without retraction. J Neurosurg Spine. 2007;6(2):121–5. 10.3171/spi.2007.6.2.121. 17330578 10.3171/spi.2007.6.2.121

[26] Lin GX, Huang P, Kotheeranurak V, Park CW, Heo DH, Park CK, Park JY, Kim JS. A systematic review of unilateral biportal endoscopic spinal surgery: preliminary clinical results and complications. World Neurosurg. 2019;125:425–32. 10.1016/j.wneu.2019.02.038. 30797907 10.1016/j.wneu.2019.02.038

[27] Song X, Hao Y, Ren Z, Yu L, Zhu G, Zhou W. Preliminary study of unilateral biportal endoscopic lumbar interbody fusion for the treatment of grade I lumbar spondylolisthesis. Chin J Minim Invasive Surg. 2022;22:814–9.

[28] Chen L, Zhu B, Zhong HZ, et al. The learning curve of unilateral Biportal Endoscopic (UBE) spinal surgery by CUSUM Analysis. Front Surg. 2022;9:873691. 10.3389/fsurg.2022.873691. Published 2022 Apr 29. 35574554 10.3389/fsurg.2022.873691 PMC9099005

[29] Kang MS, You KH, Choi JY, Heo DH, Chung HJ, Park HJ. Minimally invasive transforaminal lumbar interbody fusion using the biportal endoscopic tech- niques versus microscopic tubular technique. Spine J. 2021;21(12):2066–77. 10.1016/j.spinee.2021.06.013. 34171465 10.1016/j.spinee.2021.06.013

[30] Kang MS, Heo DH, Kim HB, Chung HT. Biportal endoscopic technique for transforaminal lumbar Interbody Fusion: review of current research. Int J Spine Surg. 2021;15(suppl 3):84–S92. 10.14444/8167. 10.14444/8167 PMC9421279 35027471

[31] Kang MS, Chung HJ, Jung HJ, Park HJ. How I do it? Extraforaminal lumbar interbody fusion assisted with biportal endoscopic technique. Acta Neuro- chir (Wien). 2021;163(1):295–9. 10.1007/s00701-020-04435-1. 10.1007/s00701-020-04435-1 32514621

[32] Abolfotouh SM, Alnori O, Choma T, Moore D, Abolfotouh MA. Epidemiology of work-related Neck Pain among Spine surgeons. Global Spine J. 2024;14(5):1515–23. 10.1177/21925682221148685. 36564909 10.1177/21925682221148685 PMC11394513

[33] Palmer KT, Smedley J. Work relatedness of chronic neck pain with physical findings–a systematic review. Scand J Work Environ Health. 2007;33(3):165–91. 17572827 10.5271/sjweh.1134

[34] Auerbach JD, Weidner ZD, Milby AH, Diab M, Lonner BS. Musculoskeletal disorders among spine surgeons: results of a survey of the scoliosis research society membership. Spine. 2011;36(26):E1715–21. 21508887 10.1097/BRS.0b013e31821cd140

[35] Wyatt RW, Lin CC, Norheim EP, Przepiorski D, Navarro RA. Occupation-related cervical spine disease in Ortho- paedic surgeons. J Am Acad Orthop Surg. 2020;28(17):730–6. 32324708 10.5435/JAAOS-D-19-00834

